# Severe hypersensitivity pneumonitis associated with everolimus therapy for neuroendocrine tumour: a case report

**DOI:** 10.1186/1756-0500-6-471

**Published:** 2013-11-18

**Authors:** Camille Sibertin-Blanc, Emmanuelle Norguet, Muriel Duluc, Guillaume Louis, Jean-François Seitz, Laetitia Dahan

**Affiliations:** 1 Assistance Publique – Hôpitaux de Marseille, Service d’oncologie digestive, Hôpital Timone, Université de la Méditerranée, Marseille, France; 2 Assistance Publique – Hôpitaux de Marseille, Service de radiologie, Hôpital Timone, Université de la Méditerranée, Marseille, France; 3 CHU Timone, 264 rue Saint Pierre, F-13385 Marseille cedex 5, France

**Keywords:** Neuroendocrine tumor, Everolimus, Hypersensitivity pneumonitis

## Abstract

**Background:**

Novel therapeutic agents are currently being investigated for neuroendocrine tumour treatment.

**Case presentation:**

We report here on the case of a patient presenting with hypersensitivity pneumonitis while being treated with everolimus, a mammalian target of rapamycin (mTOR) inhibitor.

**Conclusion:**

Side effects of everolimus should be familiar to clinicians, including nonspecialists, and be monitored carefully to allow for prompt management.

## Background

The incidence of neuroendocrine tumours is increasing. They are often diagnosed at an advanced stage, with conventional chemotherapy treatments having a limited effect. Therefore, novel therapeutic agents are currently being investigated, notably everolimus, a mammalian target of rapamycin (mTOR) inhibitor.

Neuroendocrine tumours have recently been shown to display a genetic anomaly that may lead to an activation of the mTOR pathway [1]. The mTOR kinase protein, however, is a central regulator of cellular proliferation, growth, angiogenesis, and metabolism [2]. The predominant side effects of everolimus therapy include mucositis and buccal ulcers, but less commonly, hypersensitivity pneumonitis. We report here on the case of a patient presenting with hypersensitivity pneumonitis while being treated with everolimus.

## Case presentation

A 52-year-old man was followed up in our centre for a small intestinal neuroendocrine tumour with hepatic synchronous metastases diagnosed in 2001. The patient initially underwent small bowel and hepatic surgery combined with radiofrequency ablation, the latter being repeated in 2005 for a recurrence of hepatic metastases. Due to the progression of the disease, the patient was included in the Radiant-2 [3] trial that evaluated the efficacy of octreotide plus everolimus versus placebo plus octreotide on progression-free survival (PFS); he remained in the study for a period of 2 years. Following the initial stabilisation of the hepatic lesions, the patient experienced disease progression, leading to withdrawal from the study. Unblinding revealed that the patient was in the placebo plus octreotide arm.

**Figure 1:**
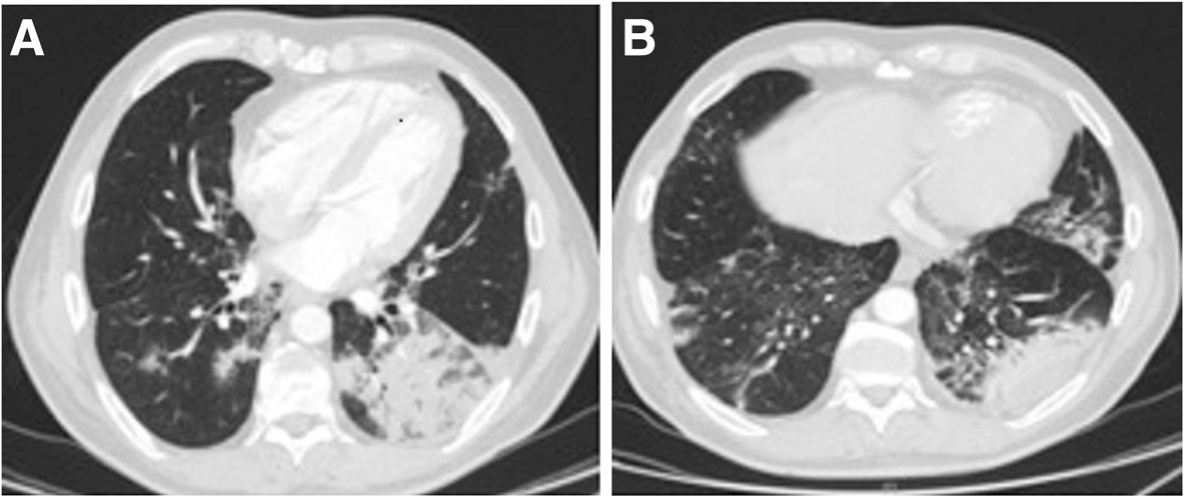
Scan taken at 4 months following the initiation of everolimus therapy during the dyspnoea episode: alveolar condensation of the right and left inferior lobe with an air bronchogram, predominant on the left-side.

**Figure 2:**
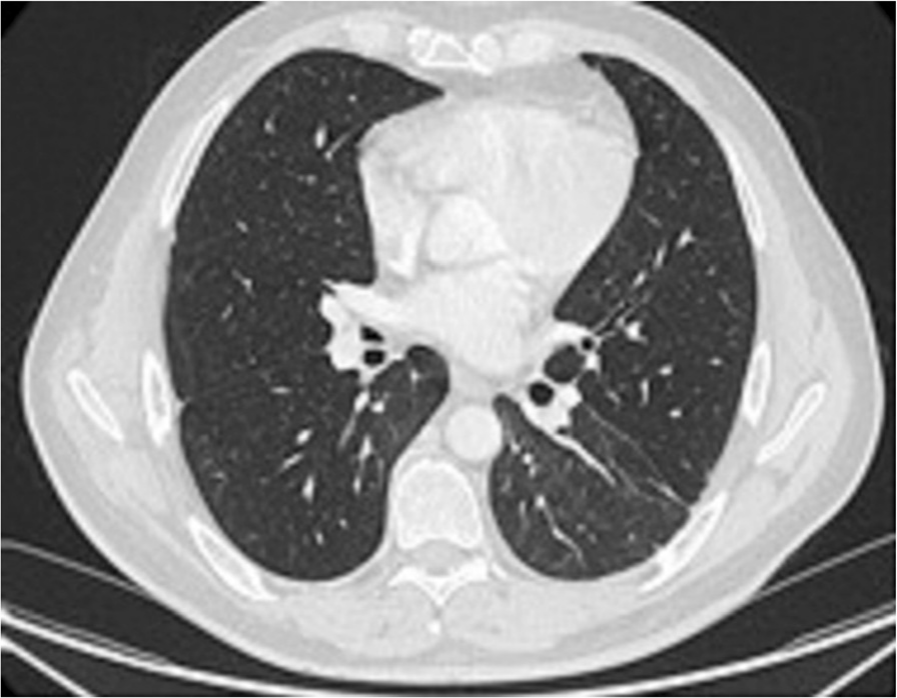
Control scan at 4 months after everolimus discontinuation: regression of bilateral parenchymatous lesions.

In February 2010, everolimus therapy was initiated. Three months later, the first evaluation revealed a partial response, with a decrease in tumour size by 28% according to RECIST (Response Evaluation Criteria In Solid Tumours) criteria. During the 4th month of treatment, the patient experienced disabling dyspnoea of grade 2, without cough. Blood gas analysis showed uncompensated respiratory alkalosis. On thoracic computed-tomography (CT) scan, there was a pattern of alveolar condensation and air bronchogram in the left and right inferior lobes (Figure 1). Pulmonary function testing revealed a moderate restrictive syndrome. The bronchoalveolar lavage (BAL) showed lymphocyte-predominant alveolitis, while bacterial screening and serology tests of atypical pneumonia were negative. It was assumed that the patient’s symptoms were due to Grade 3 hypersensitivity pneumonitis, which was likely linked to everolimus therapy. Consequently, the treatment was interrupted, following which the patient’s respiratory symptoms returned to normal as did CT images (Figure 2).

## Discussion

Everolimus is currently used in the treatment of a number of diseases: metastatic renal cancer resistant to sorafenib or sunitinib, non-small cell lung cancer unresponsive to anti-epidermal growth factor receptor (EGFR) therapy, as well as in cardiac or renal transplant patients. In addition, the drug is undergoing evaluation in metastatic breast cancer, gastric cancer, hepatocellular carcinoma, and non-Hodgkin lymphoma.

Two phase III trials assessed the efficacy of everolimus on PFS. In the Radiant-3 trial [4] conducted on patients with pancreatic neuroendocrine tumours, everolimus was associated with a significant 6.4-month increase in median PFS versus placebo. Median PFS was 11 months with everolimus plus best supportive care, compared with 4.6 months for placebo plus best supportive care (hazard ratio = 0.35; 95% Confidence Interval (CI) [0.27-0.45]). In the Radiant-2 trial [3], the improvement in PFS in patients with advanced neuroendocrine tumours just missed statistical significance at the predetermined level set by the study design, but median overall survival was higher in the everolimus arm compared with the placebo arm, 12 *vs.* 8.6 months (hazard ratio = 0.77, P = 0.026), respectively.

As described in the literature [5], the most frequently reported side-effects of everolimus include infections, oedematous syndrome, cutaneous reactions (*e.g.,* rash, itching, and dry skin), gastrointestinal problems (*e.g.*, mucositis, diarrhoea, anorexia, nausea, and vomiting), asthenia, acute renal insufficiency, and pulmonary disease, including pneumonitis as in the case of our patient. Non-infectious pneumonitis is a known class effect of rapamycin derivatives. This side-effect was found in 13.5% [5] to 25% [6] of cases in the two major everolimus efficacy trials (12% in the Radiant-2 trial and 17% in Radiant-3) and was more commonly Grade 1–2 (10-14% being Grade 1–2 and 2–3% Grade 3–4) [3,4].

Symptoms of pneumonitis include dry cough, dyspnoea, hypoxemia, asthenia, fever, and weight loss [7,8], developing between 34 and 491 days after treatment initiation [5-8]. On radiographic and CT lung imaging, infiltration or opacity of the lower lobes may be observed [6,9], even in the absence of pulmonary symptoms [5]. The BAL shows lymphocytic alveolitis, with samples testing negative for infection [7,8]. The development of this type of pneumonitis appears to be independent from serum everolimus levels [8]. The physiopathology of pneumonitis is likely based on the accumulation of collagen in the extracellular matrix, the proliferation and migration of fibroblasts, and the loss of functional alveolar gas exchange units [10].

Management depends on the severity of clinical symptoms, though the pneumonitis is usually reversible after dose reduction or treatment discontinuation [5,6,8,11,12]. In cases where the diagnosis is based on radiographic findings alone without any associated symptoms, everolimus may be continued at the same dose. In cases of moderate to severe symptoms, everolimus therapy should be temporarily stopped until pneumonitis has resolved, then re-instituted at a lower dose. Corticosteroids may also be administered [5].

## Conclusion

These relatively frequent side effects of everolimus should be familiar to clinicians, including nonspecialists, and be monitored carefully to allow for prompt management. Recommendations for optimal management strategies are necessary.

## Consent

Written informed consent was obtained from the patient for publication of this Case report and any accompanying images. A copy of the written consent is available for review by the Editor-in-Chief of this journal.

## Competing interest

The authors declare that they have no competing interests.

## Authors’ contributions

Manuscript writing: L Dahan and C Sibertin-Blanc. Review: L Dahan, C Sibertin, JF Seitz. Final responsibility for the decision to submit for publication: L Dahan. All authors read and approved the final manuscript.
